# The whole-of-society approach of mass COVID-19 vaccination in China: a qualitative study

**DOI:** 10.1186/s12961-022-00947-7

**Published:** 2022-12-30

**Authors:** Qian Wang, Zhiqiang Qu, Shiyi Tu, Xi Chen, Zhiyuan Hou

**Affiliations:** 1 School of Public Health, Fudan University, 130 Dong’an Road, Shanghai, 200032 China; 2 Department of Health Policy and Management, Yale School of Public Health, City of New Haven, United States of America; 3 Department of Economics, Yale University, City of New Haven, United States of America; 4 NHC Key Laboratory of Health Technology Assessment, Fudan University, Shanghai, China

**Keywords:** COVID-19 vaccine, Mass vaccination, Vaccination system, Whole-of-society, Qualitative study, Interview

## Abstract

**Background:**

Many countries have an inefficient vaccination system, which hinders global exit from the COVID-19 pandemic. It is vital to summarize COVID-19 vaccination practices in countries with high vaccination coverage and provide implications for other countries. This study aimed to investigate China’s COVID-19 vaccination system and to summarize its implementation experience from a health system perspective.

**Methods:**

We conducted key informant interviews in five representative cities of China in late 2021. Guided by the health systems framework proposed by WHO, we developed our interview guidelines which included seven building blocks—leadership and governance, health workforce, vaccination service delivery, vaccination mobilization and communication, financing, access to vaccines, and information systems. Semi-structured interviews and COVID-19 vaccination policy documents were collected and coded using a thematic analysis approach.

**Results:**

A total of 61 participants (nine vaccination programme directors of the local Center for Disease Control and Prevention, four government staff and 48 vaccination service workers) were interviewed. We found that China adopted a whole-of-society approach with adequate government engagement and linked health and non-health sectors to promote COVID-19 vaccination. Key measures included the collaboration of multiple systems and departments from a governance perspective, allocating sufficient health workers and resources, large-scale vaccination mobilization and communication, expansion of vaccine financing channels, localized production and digital information systems. With the vaccination system strengthening, the two-doses vaccination coverage reached 89.5% for the total population but relatively lower coverage for older adults as of July 2022.

**Conclusions:**

Our study highlights the importance of a government-led whole-of-society approach to promote mass vaccination. The low vaccination coverage among older adults should be paid the greatest attention to. The experiences and lessons from China may serve as a reference for other countries.

## Background

Mass COVID-19 vaccination has been in progress globally since the approval of COVID-19 vaccines. An efficient vaccination services delivery system and vaccine acceptance from the public are necessary to achieve high vaccination coverage. Due to the enormous target population for COVID-19 vaccination, each country faced universal challenges to relying on the current vaccination system to support mass COVID-19 vaccination. These challenges included the shortage of health workforce [1], the lack of manufacturing scalability [1, 2], inaccessibility and high cost [1, 3], and vaccine hesitancy [4–6]. Thus, it is of great importance to summarize the experiences of mass COVID-19 vaccination in countries with high vaccination coverage and help promote COVID-19 vaccination in other countries.

However, most studies investigated the acceptance and willingness of COVID-19 vaccination from the recipient’s perspective [7], and few investigated the vaccination service delivery system from the provider’s perspective. Currently, global high-income countries have made significant progress in COVID-19 vaccination, while vaccination campaigns are lagging in low- and middle-income countries [8]. Low- and middle-income countries faced greater challenges [9] due to their inefficient vaccination system, with gaps such as poor infrastructure to transport vaccines [10], limited funds and inadequate surveillance systems [11]. Previous studies have summarized the implementation strategies for COVID-19 vaccination in Israel, Italy and European countries [12–14], providing experiences and lessons from high-income countries [15, 16]. However, no vaccination experiences have been reported in low- and middle-income countries.

As of July 2022, 3.47 billion doses of COVID-19 vaccines had been administered in China, and 89.5% of Chinese had completed their initial two-dose vaccination protocol [17]. Although China has the largest population in the world, the full vaccination rate in China was higher than that in many other countries or regions, including the United States (67.0%), the United Kingdom (74.7%), Canada (82.5%), Australia (83.7%), the European Union (73.2%) and worldwide coverage (61.2%) [18]. The high vaccination rate was fuelled by the COVID-19 vaccination system in China, which has been strengthened to meet the enormous vaccination demand.

This study aimed to investigate China’s COVID-19 vaccination system and to summarize its implementation experience from a health system perspective. China’s experiences and lessons may serve as a reference for other low- and middle-income countries in promoting COVID-19 vaccination.

## Methods

### Study design

We conducted key informant interviews in five representative cities of China from late September to early November 2021. One megacity, two prefectural-level cities and two county-level cities were selected to represent different socioeconomic layers and COVID-19 epidemic status in China. The selected megacity is located in eastern China and is highly developed, with more than 15 million residents. It experienced several large-scale COVID-19 outbreaks, with more than 650 cumulative COVID-19 confirmed cases as of our study time and once had strict containment measures. The two selected prefectural-level (county-level) cities represent the urban (rural) regions in central and western China, respectively, with 1–5 (0.3–0.5) million residents in each city. The prefectural-level cities had only several small outbreaks, with 30–200 cumulative confirmed cases, whereas county-level cities rarely experienced an outbreak, with only a dozen cumulative confirmed cases. The Fudan University School of Public Health Ethics Committee approved the study protocol [FDU IRB#2018-10-0703].

### Data collection

A multistage sampling process was applied to ensure the representativeness of the sample. In each selected city, we approached its Center for Disease Control and Prevention (CDC) and selected one community-level government agency and three community health centres. We interviewed 1–2 immunization programme directors of the local CDC, one community-level government staff member who participated in the COVID-19 vaccination campaigns, and 9–12 vaccination service workers from three community health centres where vaccination sites are located. In each community health centre, we interviewed one director of the health centre, one director of the vaccination site, one physician or nurse delivering vaccination services and one physician for pre-screening and treating side-effects following vaccination.

We conducted semi-structured interviews with these key informants. Before the formal investigation, we developed interview guidelines and piloted them with 10 individuals in a non-study site. Each participant was informed of the interview purpose and was assured of voluntary participation and confidentiality. Participants signed the written consent, and we digitally recorded interviews. Among 63 individuals invited, 61 agreed to participate in the interview, including nine vaccination programme directors, four community-level government staff and 48 vaccination service workers (nine directors of community health centres, 13 directors of vaccination sites, 15 vaccination physicians or nurses, and 11 screening physicians) (Table 1). Each interview lasted around 30–60 minutes.

**Table 1:** Characteristics of study participants (*N* = 61)

Characteristics	No. (%)
City levels and locations	
Megacity in eastern region	12 (19.7)
Prefectural-level city in central region	8 (13.1)
Prefectural-level city in western region	14 (23.0)
County-level city in central region	13 (21.3)
County-level city in western region	14 (23.0)
Occupation	
Immunization programme director at CDC	9 (14.8)
Government staff at community level	4 (6.6)
Community health centre	
Director	9 (14.8)
Vaccination clinic director	13 (21.3)
Physician or nurse for vaccination	15 (24.6)
Physician for screening	11 (18.0)
Sex	
Male	16 (26.2)
Female	45 (74.8)
Age (years)	
< 30	4 (6.6)
30–40	27 (44.3)
40–50	23 (37.7)
≥ 50	7 (11.5)
Education	
High school or below	4 (6.6)
College	17 (27.9)
Bachelor’s degree	37 (60.7)
Postgraduate	3 (4.9)

In addition to key informant interviews, we collected COVID-19 vaccination policy documents at the national and provincial levels from official government websites and at the city level from local interviewed institutions. We also collected the COVID-19 vaccination data from the press conference of the Joint Prevention and Control Mechanism of the State Council of China [17] and the numbers of daily doses and cumulative doses administered per 100 people in China from the Our World in Data website [18].

### Interview guidelines

We developed our interview guidelines based on the six building blocks of the health systems framework proposed by WHO [19], including *leadership and governance*, *health workforce*, *vaccination service delivery*, *financing*, *access to vaccines* and *information system*. Unlike medical services, it is necessary to promote awareness and acceptance of vaccination as a preventive measure [20]. Therefore, we added a new building block to the original health systems framework—*vaccination mobilization and communication*.

We investigated how to strengthen the vaccination system and improve the seven health system building blocks to meet China’s massive demand for COVID-19 vaccination. *Leadership and governance* involve ensuring the existence of policy frameworks combined with effective oversight, coalition-building, regulation, attention to system design, and accountability. We focused on the overall strategy of the COVID-19 vaccination system design, oversight of vaccination campaigns, and cross-departmental accountability and collaboration in the vaccination process. For *health workforce*, we investigated how health workers were effectively allocated and trained to deliver vaccination services and treat side-effects following vaccination. For *vaccination service delivery*, we explored how to organize vaccination services to meet the needs of diverse populations, such as community residents, internal migrants and company staff. For *vaccination mobilization and communication*, we investigated how to inform the public about COVID-19 vaccines and counter misinformation to address vaccine hesitancy. For *vaccine financing*, we explored how to raise adequate funds and who paid for the COVID-19 vaccines and vaccination. For *access to vaccines*, we investigated the production, procurement and distribution of COVID-19 vaccines and consumables. For *information system*, we asked about how vaccine circulation and vaccination records were tracked and how side-effects were monitored. In each system building block, we investigated the difficulties faced and solutions in COVID-19 vaccination campaigns.

### Data analysis

Two researchers (QW and ZQ) transcribed the recordings of interviews for mutual proofreading. We applied a thematic analysis approach [21] to analyse the interview transcripts and vaccination policy documents using NVivo, version 12. We first coded our transcripts and further refined codes during the coding process. We derived common themes based on the codes across participants and synthesized them according to the seven system building blocks as mentioned above. One researcher coded all the data, and a second researcher reviewed a subset of the coding, with discrepancies discussed until reaching a consensus. When necessary, we reread the transcripts to check whether the themes produced were consistent and coherent. We also described the trends of daily doses and cumulative doses administered per 100 people.

## Results

### Leadership and governance

Efficient leadership and governance are essential for mass COVID-19 vaccination. Each interviewed city established a specific leading group to develop and coordinate local implementation strategies for COVID-19 vaccination. With effective governance, responsibilities were clearly defined for different government departments. First, the governments’ community committees shouldered the primary responsibility for vaccination mobilization and communication for community residents. Each ministry, such as the health, education and transportation ministry, was responsible for vaccination mobilization and communication for workers in the responding industry. Second, the local CDCs were responsible for supplying and distributing COVID-19 vaccines, training vaccination service workers and providing technical guidance for vaccination. Third, community health centres and hospitals undertook the primary responsibility for vaccine injection work. Vaccination sites at health centres and hospitals identified target individuals with community committees and reported the daily vaccination progress to local CDCs (Table 2).

**Table 2:** Seven building blocks of COVID-19 vaccination system strengthening in China

Building blocks	Practices for strengthening vaccination system
Leadership and governance: how to guarantee efficient leadership and clearly positioned responsibility?	
Leading body	Establish a leading group to oversee the implementation of mass vaccination, including workforce, mobilization and communication, service delivery and vaccine supply
Cross-departmental accountability	Government community committees and ministries responsible for vaccination mobilization and communication; CDCs for vaccine supply, staff training and technical guidance; community health centres and hospitals responsible for vaccination, and daily update of vaccination demand data from governments and report vaccinated amounts to CDCs
Vaccination strategy	Two-step vaccination strategy with the priority order of key groups maintaining social functions first, followed by the public
Health workforce: how to address the shortage of vaccination workforce?	
Workforce allocation	Health workers are allocated to participate in vaccination services after training and qualification exam; each vaccination site is equipped with government personnel, security and volunteers
Training	CDCs are responsible for COVID-19 vaccination training and capacity assessment for medical workers
Responsibility	Doctors for pre-check, registration and medical emergency at vaccination sites, and nurses for injection and vaccine management
Incentive	Economic and noneconomic incentives for medical workers to engage in the additional vaccination work
Vaccination service delivery: how to improve the availability of COVID-19 vaccination services?	
Vaccination sites	Traditional vaccination sites, new vaccination sites in hospitals and temporary vaccination sites as needed
Flexible services	Vaccination in health centres or hospitals for residents, door-to-door vaccination for enterprise institutions and schools, and vaccination at airport and railway stations for migrant population
Appointment	Online appointment through various platforms, appointment through community or the employer, and on-site appointment
Treatment of side-effects	Doctors from hospitals shoulder the medical emergency and treatment of side-effects following vaccination
Vaccination mobilization and communication: how to address COVID-19 vaccine hesitancy?	
Propaganda and mobilization	Rapid, large-scale and accessible publicity of COVID-19 vaccination on the internet and TV; publicity within institutions and industries by the corresponding government departments; notification by phone call and message, flyers and brochures; household screening by grassroots government staff to find the unvaccinated
Incentive	Incentives for the vaccinated such as living necessities for free or cash
Fight misinformation	Health departments identified and promptly refuted vaccine misinformation through the official websites, traditional media and social media platforms
Vaccine financing: how to raise adequate funds for the COVID-19 vaccines and vaccination?	
Free of charge	COVID-19 vaccination is free for the public; 70% of fees for COVID-19 vaccines and vaccination was directly covered by social health insurance, while the remaining was funded through reallocated general taxation
Incentive for workers	Vaccination service fees paid to workers as incentives
Access to vaccines: how to ensure adequate supply and distribution of COVID-19 vaccines?	
Procurement and distribution	Provincial CDCs responsible for unified procurement and coordinated distribution of COVID-19 vaccines
Management and inventory	Digital tracking of vaccines by scanning code for each in and out of storehouse. Daily report of the vaccinated number and inventory for each vaccination site
Supply of consumables	Vaccination sites responsible for purchasing syringes and other vaccination consumables which are paid by medical insurance funds
Information system: how to track COVID-19 vaccine circulation and side-effects?	
Vaccination tracking	Establish vaccine circulation and vaccination management information system. Each vaccine dose is tracked from production to vaccination by digital tracking code, and individual vaccination records are recorded and stored in this system
Side-effect record	Information system for recording and reporting side-effects following immunization

**Fig. 1:**
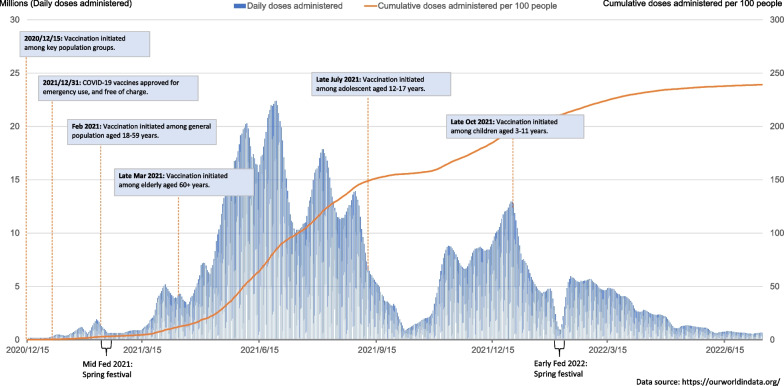
COVID-19 vaccination strategy and coverage in China. The labelled dates represent the earliest time that vaccination was initiated in a specific age group. Not all regions started vaccination at the same time, and there was also a time lag from the official announcement of vaccination to its implementation

To maintain societal and economic development, China adopted a two-step strategy for the COVID-19 vaccination rollout (Fig. 1). The first step was to implement emergency vaccination for key population groups: one group was people at high risk of occupational exposure, including health workers, community workers and those at risk of overseas infection; the other group was workers in essential positions of maintaining basic society operation such as workers engaging in national security and the production and supply of daily essentials. With the increase in vaccine supply and the updated evidence from clinical trials of COVID-19 vaccines, the vaccination strategy moved to the second step. Vaccination expanded to the public with a priority order of the 18–59 age group, individuals aged 60+ or with health conditions, the 12–17 age group and the 3–11 age group. People aged 60+ and with multiple chronic conditions were given a lower priority than those aged 18–59, partially due to the lack of inactivated vaccine trial evidence among older adults.

### Health workforce

COVID-19 vaccination targets people of all ages [22, 23]. The wide-scale target population requires a large number of vaccination workers to handle the work of pre-check, registration, injection, medical emergency and vaccine management. To address the shortage of vaccination workforce, health workers who had never engaged in vaccination services were allocated to participate in COVID-19 vaccination. Local CDCs organized training on COVID-19 vaccination, and all health workers were required to attend this online or on-site training. The COVID-19 vaccination training included preparation and implementation, operation of vaccination, treatment for suspected side-effects following vaccination, information reporting and COVID-19 vaccine management. A specific qualified vaccination certificate was offered to them after passing a qualification exam.

Those health workers needed to undertake both COVID-19 vaccination and their routine work. They often worked in shifts and would receive subsidies and incentives for the additional vaccination responsibility. Doctors mainly took responsibility for pre-check, registration and medical emergency, while nurses were primarily responsible for injection and vaccine management. The vaccination workforce in each vaccination site was adjusted according to vaccination demand. Usually, there were 15–20 vaccination workers for 3–5 vaccination units in a community health centre and around 30 vaccination workers for 8–10 vaccination units in a large temporary vaccination site to meet the extraordinary demand (Table 3). A vaccination unit includes pre-check, registration, injection and medical emergency. In addition to vaccination workers, each vaccination site was equipped with community-level government staff, security personnel and volunteers to provide assistance.

**Table 3:** Setting-up and services delivery of COVID-19 vaccination sites

Type of vaccination site	Location	Responsible organization	Vaccination unit^b^	Allocation of workers
Traditional or mobile vaccination site	Community health centre or community	Community health centre	3–5 units	(1) Around 15 health workers: two for overall administration, three for pre-check, two for registration, four for injection, two for medical emergency^a^ and two for vaccine management (2) Around two government staff at community level for the collaboration between the vaccination site and community and organizing the vaccinated individuals (3) Around two security personnel or volunteers for maintaining the order of the vaccination site
Large temporary vaccination site	Giant stadium, outdoor square, airport and railway station, hospital, etc.	Hospitals	8–10 units	(1) Around 30 health workers from hospitals: three for overall administration, seven for pre-check, six for registration, nine for injection, three for medical emergency, and two for vaccine management (2) Around two government staff at community level for the collaboration between vaccination site and community and organizing the vaccinated individuals (3) Around five security personnel or volunteers for maintaining the order of the vaccination site

^a^Doctors from hospitals are dispatched to vaccination sites for medical emergency and treatment of side-effects following vaccination

^b^A vaccination unit includes pre-check, registration, injection and medical emergency

### Vaccination service delivery

Traditionally, all vaccination sites are set up in health centres, and no vaccination services are available in hospitals in China. To improve the availability of COVID-19 vaccination services, new vaccination sites were established in hospitals and other temporary locations (Table 3). Temporary vaccination sites were set up as needed, usually located in areas with convenient transportation and concentrated populations, and were staffed by qualified vaccination facilities. In urban areas, COVID-19 vaccination sites were mainly located in community health centres, hospitals and various temporary sites. To increase vaccination convenience, some temporary vaccination sites were set up in giant stadiums or outdoor squares, and mobile vaccination buses were also used. Temporary vaccination sites were also set up in airports and railway stations for migrants. On-site vaccination services were provided for enterprises and schools. In rural areas, vaccination sites were mainly located in county hospitals, township health centres and temporary sites. Temporary vaccination sites were applied for villages far from towns, and transportation services were provided to older adults with mobility impairment.

Most vaccination sites adopted an appointment-based approach. Residents could make a vaccination appointment online or through their employer or community workers. They were also allowed vaccination without an appointment. Vaccination sites also extended working hours and even worked during holidays.

To ensure vaccination safety, each vaccination site was equipped with a vaccination space, an observation space and a space for treating side-effects following vaccination. Doctors from hospitals shouldered medical emergencies and side-effects treatment, and emergency medicines and equipment were available at each vaccination site.

### Vaccination mobilization and communication

As a newly developed vaccine at a rapid speed, public hesitancy towards COVID-19 vaccines was prevalent worldwide. To address vaccine hesitancy, the public must be informed about this new vaccine and fight vaccine misinformation. In China, government agencies such as various departments and community committees were responsible for vaccination mobilization and communication. First, COVID-19 vaccines were promoted through various online and offline channels, including official websites of the health ministry and CDC, TV news, social media platforms and all-media matrix linkages. Second, flyers, brochures, posters, audio and videos were placed at high-traffic places to popularize knowledge on COVID-19 vaccines. Third, grassroots government staff conducted door-to-door screening to find unvaccinated individuals and persuade them to get a vaccination.

In addition, health departments identified and promptly refuted misinformation and disinformation around COVID-19 vaccines through official websites, traditional media and social media platforms. Incentives were also adopted to increase the public willingness for vaccination. Daily necessities like rice, edible oil, towels, washbasins or cash were provided for free to those who received the vaccine.

### Vaccine financing

The positive externality of vaccines limits individual motivation to vaccinate. The government needs to internalize the externality by subsidizing products with positive externality, such as COVID-19 vaccines. In China, vaccines covered by the Expanded Programme on Immunization (EPI) are free of charge and fully financed by general taxes, while non-EPI vaccines are fully paid for by recipients. In early 2021, the Chinese government stipulated that COVID-19 vaccines were not included in the EPI but were free for residents. Seventy percent of fees for COVID-19 vaccines and vaccination were directly covered by social health insurance, while the remaining was funded through reallocated general taxes. This was the first time that social health insurance funds paid for vaccine fees nationally. Vaccination workers can receive vaccination fees as incentives.

The National Healthcare Security Administration adopted strategic purchasing of COVID-19 vaccines and negotiated the vaccine price with enterprises. Through several rounds of negotiations with domestic vaccine manufacturers, the purchase price of inactivated COVID-19 vaccines was set at no more than 14.2 US dollars (1 dollar = 6.36 Chinese yuan) per dose in early 2021 and further dropped to around 6.3 and 3.1 US dollars per dose in the consequent periods.

### Access to vaccines

Sufficient supply and distribution of COVID-19 vaccines can ensure access to vaccines as prerequisites of mass vaccination. China set up a national-level assurance group to promote the production of COVID-19 vaccines developed by various technical platforms. It emphasized localized production to ensure the supply and availability of COVID-19 vaccines. As the main vaccines used in China, inactivated vaccines can be transported more easily and economically than other technologies such as messenger RNA (mRNA) vaccines. A surveillance system was used to track each dose of COVID-19 vaccine. In general, provincial CDCs took responsibility for procuring and distributing COVID-19 vaccines. Provincial CDCs collected data on vaccination demand from all cities in the province, centralized procurement with vaccine manufacturers and distributed vaccines to each vaccination site. The CDC was able to rapidly adjust the distribution plans according to vaccination demand. Moreover, syringes and other vaccination consumables were purchased by vaccination sites, billed to vaccination fees and covered by health insurance funds.

### Information system

Well-functioning information systems are necessary to record vaccination status and side-effects. China digitally tracked the status of each COVID-19 vaccine dose through vaccine circulation and vaccination management information systems. A tracking code on each packaged box of the COVID-19 vaccine made it possible to track each dose by scanning the code. Through this information system, every vaccination site could identify vaccine inventory and report it to local CDCs daily.

The side-effects following COVID-19 vaccination need to be treated promptly. In China, vaccination workers must record suspected side-effects in the adverse event following immunization (AEFI) record book. Side-effects and the treatments for AEFI must be promptly reported to health administrations. Severe AFEIs require a daily report of the detailed conditions including onset, symptoms and treatment of the case.

### COVID-19 vaccination coverage

With the strengthening of the vaccination system, around 3.47 billion doses and 239 doses per 100 individuals had been administered in China as of early July 2022 (Fig. 1). According to the press conference of the Joint Prevention and Control Mechanism of the State Council of China, as of 7 July, 89.5% of the Chinese population had completed the initial two-dose vaccination protocol. However, up to 16% of older adults aged 60 years or above (42.8 million) had not completed two-dose vaccination [17].

## Discussion

This study investigated the implementation experiences of mass COVID-19 vaccination in China according to the WHO health systems framework [19]. China adopted a whole-of-society (WoS) approach with adequate government engagement to promote mass vaccination. The key measures in this WoS approach included the collaboration of multiple systems and departments from a governance perspective, allocation of sufficient health workers and resources, large-scale vaccination mobilization and communication, expansion of vaccine financing channels, localized production and use of a digital information system. These measures accelerated and achieved mass vaccination in China and could provide evidence for promoting mass vaccination in countries with low vaccination coverage.

WHO proposed the WoS governance system as guidance for pandemic preparedness and response [24]. It requires the cooperation of multiple systems and multiple sectors and has been widely used for health policy-making [25–28]. China adopted the principle that “governments undertake mobilization and health sectors deliver vaccination”, with all ministries jointly promoting COVID-19 vaccination. China’s COVID-19 vaccination system involved the health sector and various essential service sectors including transport, security, publicity, volunteers, finance and social organizations. These health and non-health sectors collaborated to engage in almost all parts of COVID-19 vaccination, including vaccination workforce allocation, access to vaccines, and vaccination mobilization and communication. The successful implementation of the WoS approach might be facilitated by the centralized administration system in China [29, 30]. It can enable a top-down leadership mechanism, multisystem collaboration and consistency in policy implementation from the central to local [30, 31]. Government-led WoS governance has contributed to mass COVID-19 vaccination in China. It may provide an approach to coordinate various entities of society as a whole to promote mass vaccination.

Vaccination resources are usually in severe shortage when facing mass vaccination. To meet the enormous demand for COVID-19 vaccination, China adjusted its traditional vaccination system with dedicated vaccinators and vaccination sites. Before COVID-19 vaccination campaigns, vaccination services were only delivered by dedicated vaccinators at community health centres [32]. There were only 3–5 vaccinators in a community health centre, and they could not manage to serve tens of thousands of residents. Therefore, many health workers in community health centres and hospitals were widely mobilized to receive training and dispatched to deliver COVID-19 vaccination services. Temporary vaccination sites and on-site vaccination services were also established. These activities were also found in Israel [33–35]. The engagement of more health workers and the expansion of vaccination sites have greatly improved the convenience and access to vaccines. However, vaccination services have yet to be included in their routine work and responsibilities, especially for general practitioners, which hindered the sustainability of COVID-19 vaccination. China should permit general practitioners to deliver vaccination services routinely.

COVID-19 vaccine misinformation and hesitancy were prevalent globally [6, 36], and as a result, vaccination mobilization and communication became the key to addressing vaccine hesitancy. All departments and levels of government in China engaged in the mobilization and communication of COVID-19 vaccination. Its success depends on public trust in the government [37]. Previous studies confirmed the high support of the Chinese for COVID-19 mitigation measures such as mask-wearing and COVID-19 vaccination [38, 39]. Note that in China, older adults had worse health literacy and were more vulnerable to misinformation than other age groups [40]. Therefore, older adults should become the priority group for vaccination mobilization and communication. Overall, community-based publicity and mobilization could serve as an efficient strategy to decrease vaccine hesitancy and promote vaccination [41].

With the vaccination system strengthening, the two-dose vaccination coverage reached nearly 90% as of July 2022. However, among older adults aged 60+, only 83.8% had completed the initial two-dose vaccination, and only 65.7% had received a booster dose [17]. Low vaccination coverage would put older adults at risk of severe illness or death from COVID-19 infection. In China, poor vaccination coverage among older adults was also found for general vaccines such as influenza and pneumonia vaccines [40]. Before the COVID-19 pandemic, vaccines were usually self-paid for older adults, and there was a lack of vaccination publicity, leading to low awareness and coverage of general vaccines [42]. Older adults usually have more concerns about vaccine safety than other age groups [40]; however, ambiguous statements about contraindications in China’s COVID-19 vaccination guidelines exacerbated this concern and made vaccinators reluctant to serve older adults, especially those with underlying diseases [42]. More importantly, China placed older adults as a low priority for COVID-19 vaccination, in contrast to WHO recommendations and western countries, which prioritized older adults [43]. Insufficient vaccination among older adults would be a disaster when facing the more contagious Omicron variant. Therefore, China should promote COVID-19 vaccination among older adults as the highest priority.

Another challenge was the fatigue among vaccination workers. Hefty workloads with extended working hours have caused fatigue, exhaustion and psychological disturbances [44]. It is essential to offer economic and noneconomic incentives for vaccination workers to ensure their enthusiasm [45]. Arranging reasonable working hours might be vital to their efficiency and mental status [44].

We acknowledge that there may be limitations in our findings. First, qualitative interviews introduced a degree of unavoidable subjectivity and limited extrapolation, although we interviewed people at different levels of the public health system and in different geographic areas. There may be some bias since participants were chosen to be interviewed because of their position and expertise. Second, the vaccination coverage data for the studied cities were unavailable, so we cannot differentiate the performance of local vaccination campaigns. Third, this study focused on the supply side of vaccination campaigns, which complements our understanding based on previous studies on the demand side.

## Conclusion

Our study summarized China’s practices from seven building blocks of the COVID-19 vaccination system according to the experiences of workers and stakeholders working in various organizations. It highlights the importance of a WoS approach with adequate government engagement to promote mass vaccination. The low vaccination coverage among older adults should be given the greatest attention. The experiences and lessons from China may serve as a reference for other countries, especially for low- and middle-income countries, with adaptations to local contexts.

## Data Availability

The data used in this study are available from the corresponding author on reasonable request.
